# Anti-*Escherichia coli O157:H7* Properties of Purple Prairie Clover and Sainfoin Condensed Tannins

**DOI:** 10.3390/molecules18022183

**Published:** 2013-02-08

**Authors:** Xiu-Li Liu, Yong-Qing Hao, Long Jin, Zhong-Jun Xu, Tim A. McAllister, Yuxi Wang

**Affiliations:** 1 Department of Veterinary Medicine, Inner Mongolia Agricultural University, Hohhot 010020, China; E-Mails: shiguangnmg@163.com (X.-L.L.); yongqinghao1960@yahoo.com.cn (Y.-Q.H.); 2 Lethbridge Research Centre, Agriculture and Agri-Food Canada, P.O. Box 3000, Lethbridge, AB T1J 4B1, Canada; E-Mails: long.jin@agr.gc.ca (L.J.); zhongjun.xu@agr.gc.ca (Z.-J.X.); tim.mcallister@agr.gc.ca (T.A.M.)

**Keywords:** condensed tannins, *E. coli*, outer membrane, cell aggregation, protein precipitation

## Abstract

Condensed tannins (CT) from purple prairie clover (PPC; *Dalea purpurea* Vent.) and sainfoin (SF; *Onobrychis viciifolia*) were assessed for anti-*Escherichia coli* activity by comparing their ability to react with proteins and liposome, cause cell aggregation, and alter outer membrane (OM) morphology and permeability. The PPC CT had greater (*P <* 0.01) protein-precipitating capacity than SF CT using either bovine serum albumin or ribulose 1,5-disphosphate carboxylase as model proteins. Minimum inhibitory concentration of PPC CT for two strains of *E. coli* and five strains of *E. coli* O157:H7 was four to six times lower than that of SF CT. *E. coli* exposed to 10 µg/mL of both CT had higher (*P <* 0.05) OM permeability than controls and was greater (*P <* 0.05) for PPC than for SF CT. Addition of both CT at 50 and 200 µg/mL caused cell aggregation which was more evident (*P <* 0.05) for PPC than for SF CT. Transmission electron microscopy showed electron dense material on the cell surface when cells were exposed to 50 µg/mL of PPC CT. The greater anti-*E. coli* activity of PPC than SF CT was due to its enhanced ability to precipitate protein that increased OM permeability and promoted cell aggregation.

## 1. Introduction

Ruminants are a major reservoir of *Escherichia coli* serotype O157:H7, a food-borne pathogen that costs millions of dollars annually in food processing and health care in North America. Decreasing the level of *E. coli* O157:H7 in cattle may reduce the contribution of this pathogen to foodborne illness [1,2], but at present there are no effective means to reduce the prevalence of *E. coli* O157:H7 in the digestive tracts of cattle [3]. Some plant tannins have been shown to possess anti-*E. coli* O157:H7 activities and their efficacy depends on the tannin sources [4,5,6]. 

Purple prairie clover (PPC; *Dalea purpurea* Vent.) and sainfoin (SF; *Onobrychis viciifolia*) are legume forages that contain high concentrations (58–94 g/kg DM) of condensed tannins [7,8]. However, condensed tannins (CT) or proanthocyanidins isolated from PPC possess stronger anti-*E. coli* O157:H7 activity *in vitro* than CT isolated from SF [6]. The reason why these two CT differ in their inhibition of *E. coli* and *E. coli* O157:H7 is unknown. Condensed tannins are secondary plant compounds that defend plants against attack from insects and pathogens [9]. Anti-microbial activity of CT against gram-positive bacteria has been reported to be greater than against Gram-negative bacteria [10,11,12]. We have previously identified the cell membrane as the primary site that CT act upon bacteria [5,11]. The reduced activity of CT against Gram-negative bacteria has been attributed to the outer membrane (OM) structure of the bacteria [10,13]. We hypothesize that the ability of CT to inhibit the growth of *E. coli* and *E. coli* O157:H7 is due to the disruption of OM function. Proteins and lipids are important components of the cell membrane of gram-negative bacteria. Condensed tannins readily form complexes with proteins, with the degree of affinity dependent on their chemical structures [14]. Catechins, the CT monomers in green tea (*Camellia sinensis*) have been reported to express anti-*E. coli* activity by damaging the lipid bilayer and increasing OM permeability [10,15]. Other plant bioactives such as essential oil from *Melaleuca alternifoli*, thymol, eugenol, cinnamaldehyde and phenolic extracts from raspberry (*Rubus idaeus*, var. Ottawa) and cloudberry (*R. chamaemorus*) have also been shown to exhibit activity against Gram-negative bacteria by altering OM and lipid composition [16,17,18]. However, the mechanism whereby CT alter membrane function in *E. coli* is not understood. The objectives of this study were to determine the reactivity of PPC and SF CT with protein and liposome and their minimum inhibitory concentrations against *E. coli* and *E. coli* O157:H7 and to assess their effects on the outer cell membrane. 

## 2. Results

### 2.1. Protein-Precipitating Capacities of PPC and SF CT

**Figure 1:**
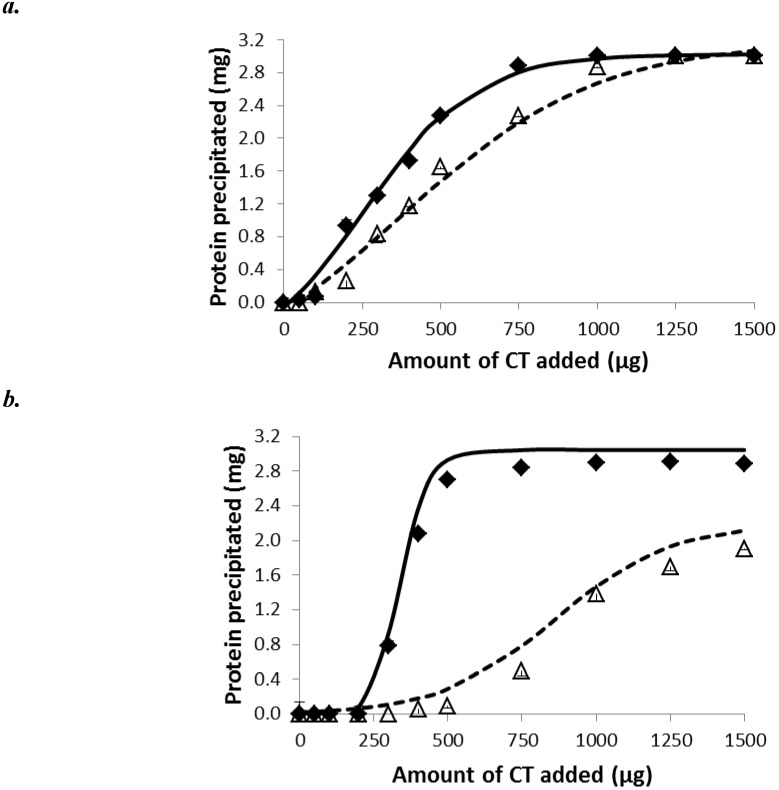
Precipitation of bovine serum albumin (BSA, *a*) and spinach ribulose 1, 5-disphosphate carboxylase (Rubisco; *b*) by condensed tannins (CT) of purple prairie clover (PPC; *Dalea purpurea* Vent.) and sainfoin (SF; *Onobrychis viciifolia*). ♦ PPC CT; ∆ SF CT. Solid and dash lines relate to PPC and SF CT data, respectively which were fitted to sigmoidal curve as described in the text. Bars indicate standard error.Where not visible, bars fall within symbols.

Protein precipitation data fitted to the sigmoidal equation* y=a_0_*+*a/*(*1+exp*(*-*(*x-b*)*/c*)) (R^2^=0.997±0.002). Bovine serum albumin (BSA) was completely precipitated by ≥750 µg of PPC and ≥1,250 µg of SF CT (Figure 1). In contrast, ribulose 1,5-disphosphate carboxylase (Rubisco) protein was completely precipitated by ≥500 µg PPC CT, whereas only 73% (2.2/3.0) of Rubisco was precipitated by SF CT at the highest concentration tested (1,500 µg). Condensed tannins from PPC exhibited a greater ability (lower protein-precipitating capacity; *PP*) to bind both BSA and Rubisco protein (*P <* 0.001) than SF CT with this response being accentuated for Rubisco (Table 1).

**Table 1:** Parameters of precipitating capacities of purple prairie clover (PPC; *Dalea purpurea* Vent.) and sainfoin (SF; *Onobrychis viciifolia*) condensed tannins (CT) with bovine serum albumin (BSA) and ribulose 1, 5-disphosphate carboxylase (Rubisco) protein.

Proteins	Parameters *	PPC	SF	SEM ^ǂ^	*P* values
BSA	*a_0_+a*	3.0	3.0	0.01	0.213
	*b*	258	368	30.0	0.584
	*c*	176	316	9.9	<0.001
	*PP*	233	364	9.4	<0.001
Rubisco	*a_0_+a*	3.0	2.2	0.02	<0.001
	*b*	335	863	22.1	<0.001
	*c*	50	195	13.4	0.002
	*PP*	299	852	16.4	<0.001

* Parameters were obtained by fitting the amount of precipitated protein (mg) and amount of CT (µg) incubated with equation: *y* = *a_0_*+*a/(1+exp(-(x-b)/c))*, where *y* = mg of protein (BSA or Rubisco) precipitated , *x* = µg of CT incubated, *a_0_+a* = estimated maximal amount (mg) of protein (BSA or Rubisco) precipitated, *b* = sigmoidal centre (µg of CT at the 50% of maximal protein precipitation), and *c* = sigmoidal width; *PP*: Protein-precipitating capacity; expressed as µg CT required to precipitate 1 mg of protein (N = 12); ^ǂ^ SEM, standard error of the mean.

### 2.2. Reactivity of PPC and SF CT with Liposome

Liposome alone and phosphate buffer containing 1-*N*-phenylnaphthylamine (NPN) with various concentrations of CT had negligible relative fluorescence value (RFV; data not shown). However, addition of NPN to liposome resulted in the highest RFV (0 CT; Table 2). The RFV linearly decreased (*P <* 0.001) with increasing concentrations of CT from 0 to 200 µg/mL. The RFV of liposome + NPN was reduced (*P <* 0.01) with the addition of PPC and SF CT, but did not differ between the two sources of CT. Fluorescence microscopy showed that the liposome aggregated upon the addition of both CT (data not shown). 

**Table 2:** Relative fluorescence values of liposome and 1-*N*-phenylnaphthylamine (NPN) mixtures after addition of purple prairie clover (PPC; *Dalea purpurea* Vent.) and sainfoin (SF; *Onobrychis viciifolia*) condensed tannins (CT).

Time	Source	CT concentration (µg/mL)				SEM *	*P* value (L ^ǂ^)
		0	10	50	200		
10 min	Control	286				5.7	
	PPC		207	184	178		<0.001
	SF		203	195	168		<0.001
30 min	Control	257				7.8	
	PPC		198	183	167		<0.001
	SF		191	190	166		<0.001

* SEM, standard error of the mean; ^ǂ^ Linear effect of CT.

### 2.3. Minimum Inhibitory Concentration (MIC) of the CT

The selected *E. coli* and *E. coli* O157:H7 strains had relatively similar sensitivity to both CT. For all strains, PPC CT completely inhibited bacterial growth at the concentrations as low as 20 µg/mL (Table 3). In contrast, the MIC for SF CT was higher, ranging between 100 and 150 µg/mL. Among the selected strains, ATCC 25922, H4420N and E318N varied in their sensitivities to both CT across assays. 

**Table 3:** Minimum inhibitory concentration (MIC) of condensed tannins (CT) of purple prairie clover (PPC; *Dalea purpurea* Vent.) and sainfoin (SF; *Onobrychis viciifolia*) to *Escherichia coli* and *E. coli* O157:H7.

Species	Strains	MIC (CT µg/mL)	
		PPC	SF
*E. coli*	ATCC 25922	20–40	100–150
	ATCC 35352	20	125
*E. coli* O157:H7	3081	20	100
	H4420N	20–50	120
	E318N	20–30	110
	EDL933	20	120
	R508N2006	20	110

### 2.4. Effects of CT on OM Permeability and Cell Aggregation of *E. coli*

**Figure 2:**
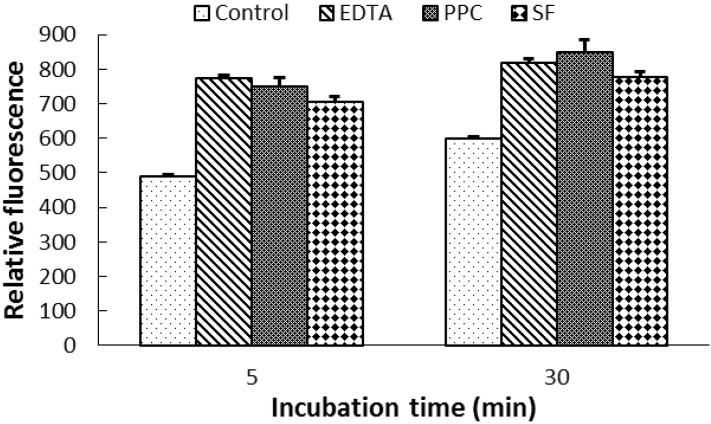
1-*N*-Phenylnaphthylamine (NPN) fluorescence of *Escherichia coli* (ATCC25922) pre-cultured with ethylenediaminetetraacetic acid (EDTA; 0.05µmol/mL) or condensed tannins (10 µg/mL) from purple prairie clover (PPC; *Dalea purpurea* Vent.) and sainfoin (SF; *Onobrychis viciifolia*). Bars indicate standard error.

Pre-culturing *E. coli* strain 25922 with 10 µg/mL of PPC or SF CT or 0.05 µmol/mL of ethylenediaminetetraacetic acid (EDTA) increased (*P <* 0.01) OM permeability as indicated by the increased RFV as compared to that pre-cultured without CT and EDTA (Control; Figure 2). The two CT sources did not differ in their impacts on OM permeability as indicated by the similar RFV of *E. coli* pre-cultured with two sources of CT.

**Figure 3:**
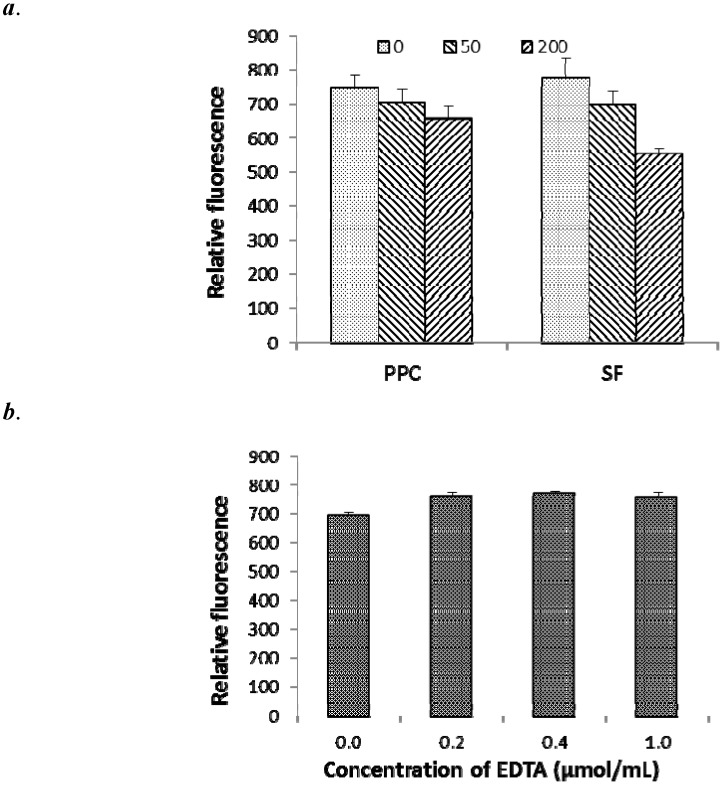
Quenching of 1-*N*-phenylnaphthylamine (NPN) fluorescence by addition of condensed tannins (0, 50 and 200 µg/mL; *a*) from purple prairie clover (PPC; *Dalea purpurea* Vent.) and sainfoin (SF; *Onobrychis viciifolia*), in comparison with addition of ethylenediaminetetraacetic acid (EDTA; *b*), to *Escherichia coli* (ATCC 25922) suspension prepared from non-exposed bacteria. Bars indicate standard error.

Addition of two CT at 50 and 200 µg/mL to *E. coli* pre-cultured without CT caused cell aggregation as indicated by decreased (*P <* 0.01) RFV, whereas addition of EDTA at the levels of 0.2, 0.4 and 1.0 µmol/mL to the same *E. coli* pre-cultured without CT slightly (*P =* 0.07) increased the RFV (Figure 3). The reduction of RFV by addition of CT was also observed for *E. coli* pre-cultured with 10 µg/mL of CT (data not shown).

**Figure 4:**
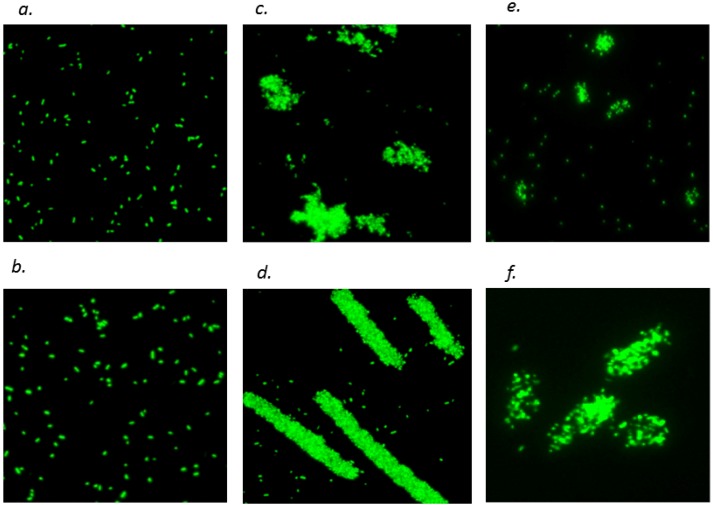
Aggregation of *Escherichia coli* (strain 25922) cell in the presence of condensed tannins (CT) of purple prairie clover (PPC; *Dalea purpurea* Vent.) and sainfoin (SF; *Onobrychis viciifolia*). *a* and *b*, bacterial suspensions prepared from culture pre-incubated with 0 and 10 µg/mL of PPC CT for 10 h; *c* and *d*, bacterial suspensions added with 50 and 200 µg/mL of PPC CT; *e* and *f*, bacterial suspensions added with 50 and 200 µg/mL of SF CT.

Fluorescence microscopy showed that *E. coli* pre-cultured without CT were evenly suspended in buffer (Figure 4*a*), and aggregation was not obvious at 10 µg/mL of PPC CT (Figure 4*b*) or 10 µg/mL of SF CT (data not shown). However, addition of 50 and 200 µg/mL of PPC and SF CT dramatically increased cell aggregation (Figure 4*c*, *d*, *e* and *f*), which was more apparent at 200 than at 50 µg/mL (Figure 4*d* & *f vs. c* & *e*) for both CT (Figure 4*c* & *d vs. e* & *f*).

### 2.5. Morphological Alteration of Cell Membrane

**Figure 5:**
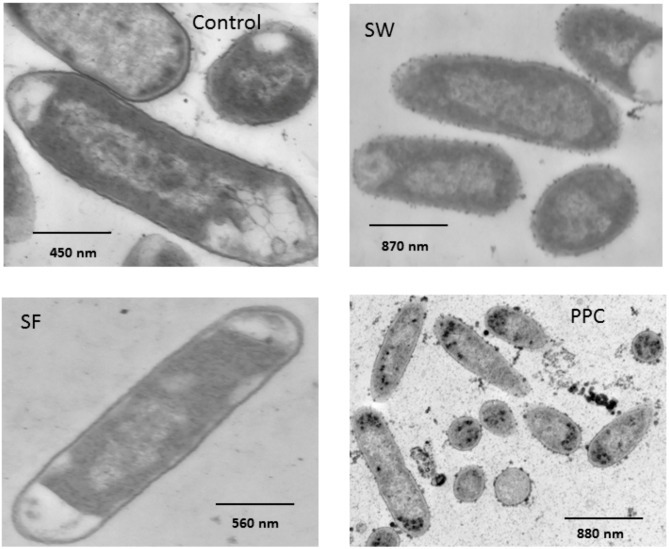
Effect of tannins isolated from purple prairie clover (PPC; *Dalea purpurea* Vent.), sainfoin (SF; *Onobrychis viciifolia*) and brown seaweed (SW; *Ascophyllum nodosum*) on cell membrane of *Escherichia coli* (strain ATCC 25922). *E. coli* was aerobically cultured in M9 media containing no tannins (Control) or containing 50 µg of condensed tannins isolated from PPC or SF or 25 µg/mL phlorotannins from SW for 12 h.

Transmission electron microscopy (TEM) examination showed that tannins acted primarily on the OM of the bacterial cell (Figure 5). 

**Figure 6:**
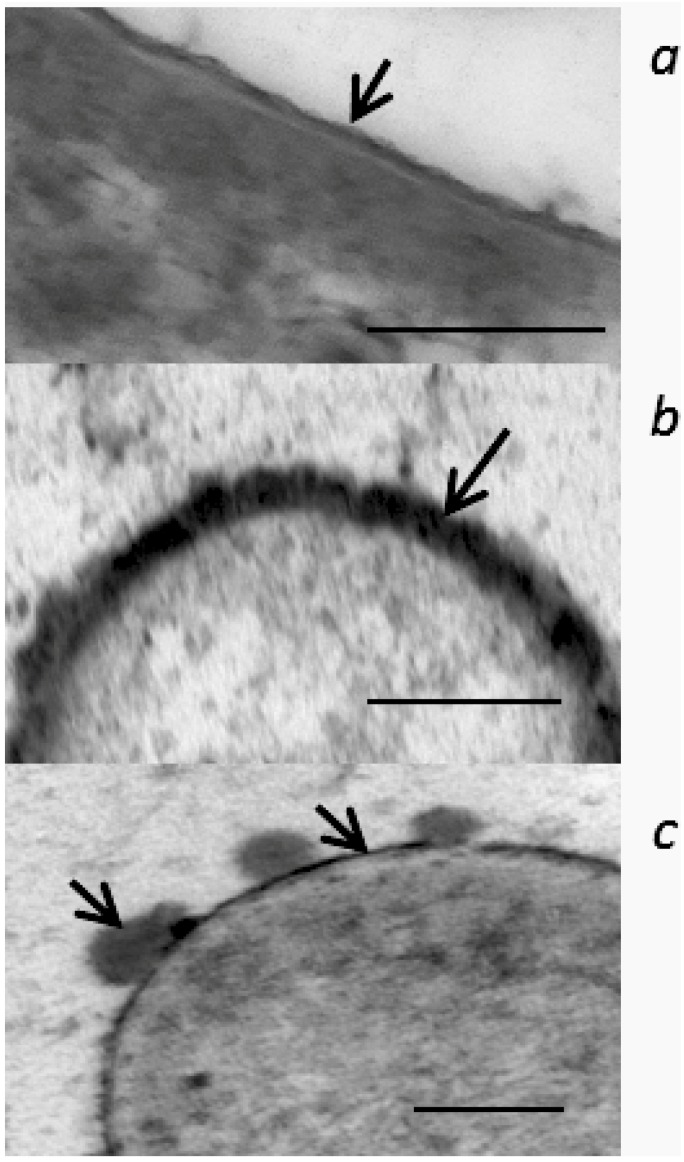
Effect of CT isolated from purple prairie clover (PPC; *Dalea purpurea* Vent.) on OM of *Escherichia coli* (strain ATCC 25922). *E. coli* was aerobically cultured in M9 media containing 0 (*a*) or 50 µg/mL of CT (*b*, *c*) for 12 h. The arrow shows OM of *E. coli* incubated without CT (*a*) and OM that was affected by PPC CT at the concentration of 50 µg/mL (*b*, *c*). Length of bar in each graph indicates 100 nm.

A continuous OM was observable in bacteria cultured without tannins and with 50 µg/mL of SF CT, although this exposure has slightly thickened this structure. In contrast, cells exposed to PPC CT and phlorotannins isolated from brown seaweed (SW) exhibited electron dense deposits on the cell surface, but cell lysis was not evident. The electron dense deposits caused by CT either continuously surrounded the cell surface (Figure 6*b*), aggregated into small droplets on the cell surface or within the vicinity of the cell (Figure 6*c*).

## 3. Discussion

The four to six times lower MIC for PPC CT compared to that of SF CT (Table 3) indicated that PPC CT possesses stronger anti-*E. coli* and anti-*E. coli* O157:H7 activity. This is consistent with our previous findings [6]. However, the reason why these two CT possess different anti-*E. coli* activities is unknown. This study revealed that different protein-precipitating capacities of the two CT may in part be responsible for their differences in anti-*E. coli* activity. It needs to be pointed out that fitting data of Rubisco protein precipitation to sigmoidal curve was not as good as that of BSA. The reason for this is unknown; however, the difference between the two CT in their capacities to precipitate Rubisco protein was even larger than to precipitate BSA. Binding and precipitating proteins is a common characteristic of tannins and it is generally proposed to be responsible for their biological activities [14]. The OM of *E. coli* is rich in protein [19,20]. Consequently, it is not surprising that the CT in our study exhibited a high affinity for the OM of *E. coli*. McAllister *et al.* [21] reported that among nine forage CT examined SF CT had the lowest protein-precipitating capacity as well as the lowest activity against the gram-negative rumen bacterium, *Fibrobacter succinogenes*. Shimamura *et al*. [22] demonstrated that the antimicrobial activity of tea catechins, a structural component of CT, is mainly attributable to the direct binding to peptides of bacterial origin. It is generally regarded that formation of CT-protein complexes depends on structure and composition of both CT and protein. For a given protein, the number of potential hydrogen and hydrophobic bonding sites on the CT molecules determines the extent of their capacities to bind protein. The number of potential hydrogen bonding sites on a given molecule of CT is a function of the number and type of monomeric subunits (e.g., flavan-3-ols), stereochemistry, and the carbon-carbon linkages between them that create linear and /or branching polymers. The SF CT have been shown a highly complex mixture of varying molecular weights [23,24]. However, there is no information on the chemical structure and composition of PPC CT. Further research in this area is needed to fully understand the anti-*E. coli* and anti-*E. coli* O157:H7 activity of PPC CT. 

Although tannins are well known for their capacities to prevent peroxidation of lipids there is little information regarding the reactivities of CT with lipids. This study showed that both PPC and SF CT promote aggregation of liposome composed of L-α-phosphatidylcholine (Table 2) as indicated by the decrease of RFV of liposome suspension upon addition of CT. Catechins from green tea have also been reported to cause aggregation of liposomes [10,15]. It has been shown that catechins are less likely to cause aggregation of liposome with a negative surface charge [10]. This may partially contribute to the lower sensitivity of Gram-negative bacteria to tannins than that of Gram-positivebacteria [10]. It is also evident that tannic acids react with choline moieties in phospholipids such as phosphatidylcholine [25]. However, the fact that PPC and SF CT aggregated liposome to a similar extent suggests that these two CT have similar reactivity with L-α-phosphatidylcholine. Therefore the contrasting anti-*E. coli* activities of PPC and SF CT are not likely due to their abilities to react with microbial lipids.

The OM of *E. coli* consists of a lipid bilayer structure composed of an outer layer of lipopolysaccharide (LPS) and proteins and an inner layer composed of phospholipids, which acts as a barrier to hydrophobic antibiotics, bile salts, detergents, proteases, lipases and lysozyme [20]. Measuring NPN uptake by cell is a common method of assessing its OM permeability [10,26]. The increased RFV observed for *E. coli* cultured with 10 µg/mL of CT (Figure 2) indicates that these phenolics at this concentration increased the OM permeability of *E. coli*. The results that PPC CT increased OM permeability and inhibited *E. coli* to a greater extent than SF CT suggest that the alteration of OM permeability contributed to anti-*E. coli* properties of CT. Increased OM permeability has been reported for other antimicrobials including essential oils [27], chitosan [28], citric acid [29] and peptides [26]. It is possible that the reaction of CT with protein and phospholipid in OM disrupted the integrity of this structure in a manner that increased its permeability. EDTA, a divalent cation chelator, is regarded as a potent OM permeabilizer [30]. Therefore, the concomitant increase in OM permeability by both EDTA and CTs used in this study may indicate that the enhanced OM permeability by CT may have also involved the chelation of cations, a possibility that is supported by the fact that chelating of metal ions has been shown to be one of the antimicrobial properties of tannins [31].

The decreased RFV of cell suspension upon addition of 50 and 200 µg/mL CT (Figure 3) is likely a reflection of cell aggregation rather than a decrease in OM permeability. This is supported by the results from fluorescence imaging analysis of this study (Figure 4*a*). Others have shown that tannins cause aggregation by increasing hydrophobicity of the cell surface [32,33], a response that has also been reported for catechins [10,15]. In contrast, increasing concentrations of EDTA increased RFV (Figure 4*b*), which is consistent with an increasing affinity for membrane cations. Therefore, at high concentrations of CT, the NPN assay may underestimate the impact of CT on OM permeability due to increased cell aggregation. 

The electron dense deposits on the cell surface observed within the *E. coli* treated with PPC CT and phlorotannins (Figure 5) are likely complexes of tannins with protein and lipids of the OM especially those contained in outer leaflet since this study showed PPC CT precipitated and aggregated both protein and liposome. It is known that some of the proteins produced within plasma are translocated across OM as extracellular proteins [19] and *E. coli* secrete OM micro-vesicles that are composed of protein, lipids and LPS similar to OM [34]. Therefore, PPC CT may also react with them to form the electron dense deposits on the cell surface as well as in the medium. These observations clearly demonstrated that tannins primarily act on OM, and that the greater anti-*E. coli* activity of PPC CT is associated with their greater capacities to perturb OM function by reacting with proteins than that of SF CT. The OM has been implicated in conjugation between bacterial cells as well as in the adhesion mechanism between bacteria and their host organism. In this regards, the disruption of the OM structure and function by CT may affect bacterial colonization capacity. Research is needed in this area. 

## 4. Experimental

### 4.1. Isolation and Purification of PPC and SF CT

Condensed tannins from PPC and SF were extracted and purified as described by Wang *et al.* [5]. Purified CT were stored in a sealed amber glass bottle at −20 °C and the same batch of each CT was used for all experiments in this study. The CT solutions were freshly prepared by dissolving CT into sterilized distilled water for each assay immediately prior to use. 

### 4.2. Determination of the Protein-Precipitating Capacities of PPC and SF CT

The protein-precipitating capacities of PPC and SF CT were determined using a modification of the procedure described by McAllister *et al*. [21]. Bovine serum albumin and ribulose 1,5-disphosphate carboxylase (Rubisco; MW 557 kDa) E.C. 4.1.1.39 isolated from spinach (Sigma-Aldrich, St. Louis, MO, USA) were used as model proteins. The BSA was dissolved (3 mg/mL) in 0.2 M acetate buffer (pH = 5.0) containing 0.17 M NaCl, and the Rubisco was dissolved (4 mg/mL) in 1 M 2-amino-2-(hydroxymethyl)-1,3-propanediol hydrochloride (Tris HCl; pH = 7.8). One milliliter of either protein solution was combined with 0.5 mL of aqueous solutions containing 0, 50, 100, 200, 300, 400, 500, 750, 1000, 1250 or 1500 µg of CT from each source. Each mixture was vortexed, allowed to stand at room temperature for 30 min, then centrifuged (15,600 × *g*, 10 min). A subsample (1.0 mL) of the supernatant was then added to 0.5-mL of aqueous polyethylene glycol (Sigma, MW 3,350; 12 mg/mL), vortexed and centrifuged (15,600 × *g*; 10 min) after standing at room temperature for 30 min. Protein remaining in solution was quantified colorimetrically using a Dye Reagent Concentrate Kit II (Catalog number 500-0002; BioRad Laboratories, Mississauga, ON, Canada) and compared to an original freshly prepared solution of BSA or Rubisco. Each assay consisted of nine replicates for each dose of CT and the assay was repeated three times over a 1-week period. 

### 4.3. Determination of Reactivity of PPC and SF CT with Liposome

The reactivity of both CT with liposome was assessed by determining their capacities in causing liposome aggregation. L-α-phosphatidylcholine (MW 768.1) from egg yolk (Sigma; ≥99%) was used as model liposome. Liposome was first dissolved into chloroform (Aldrich Chemical, USA), then dried under N_2_ gas at room temperature and the residue was subsequently re-suspended into phosphate buffer (0.2 mM, pH = 7.2) stock solution to a concentration of 7.5 µmol/mL. Four stock suspensions were prepared for each determination. 

The assay procedure was similar to that described by Ikigai *et al.* [10] to determine CT-caused liposome aggregation by measuring quenching of 1-*N*-phenylnaphthylamine (NPN; Sigma-Aldrich) fluorescence by addition of CT. The NPN is an uncharged lipophilic dye with weak fluorescence in aqueous environments but exhibits enhanced fluorescence in nonpolar or hydrophobic environments such as liposome. The assay was conducted with black fluorotitre microplates (COSTAR^®^, Corning, NY, USA), and fluorescence was measured using a fluorescence spectrophotometer (Multi-Reader, Thermo, Appliskan, Vantaa, Finland). Excitation and emission wavelengths were set at 355 and 420 nm, respectively. Briefly, liposome suspension (3.0 mL) and 1.0 mM NPN solution (3.0 µL) were mixed in 15-mL glass tubes, and then 190 µL of the mixture was transferred into black fluorotitre microplate well to which 10 µL of each CT solution was added (final CT concentration 0, 10, 50 or 200 µg/mL). In addition, two sets of glass tubes, one with 3.0 mL of liposome suspension plus 3.0 µL of phosphate buffer and another one with 3.0 mL of phosphate buffer plus 3.0 µL of NPN solution (final NPN concentration 1.0 µM) and 10 µL of each respective CT solution (final CT concentration 0, 10, 50 and 200 µg/mL) were prepared in parallel to estimate background fluorescence. A total of four replicates were conducted for each sample and the experiment was repeated three times with freshly prepared liposome suspensions. The effect of CT on liposome aggregation was assessed by comparing the RFV of liposome suspensions with different CT concentrations. In addition, the aggregation of liposome caused by the PPC and SF CT was also visually observed and photographed using LIVE/DEAD® *Bac*Light Bacterial Viability kit (L7007, Molecular Probes, Eugene, OR, USA) and fluorescence microscopy.

### 4.4. Minimum Inhibitory Concentration (MIC) of CT

#### 4.4.1. Pre-incubation of *E. coli* and *E. coli* O157:H7

Two generic *E. coli* strains (ATCC 25922 and ATCC 35352) and five *E. coli* O157:H7 strains (3081, H4420N, E318N, EDL933 and R508N2006) were used in this experiment. Bacteria were grown to log phase in 50 mL of M9 medium (M9 Minimal Salts, Sigma) at 37 °C for 10 h. The culture was then centrifuged (7,000 × *g*, 10 min), washed with 5 mL of sterilized 0.9% (w/v) NaCl solution, centrifuged (7,000 × g, 10 min) and the bacterial pellet was re- suspended in 0.9% NaCl solution. The suspension was subsequently adjusted to OD_420_ of 1.0. Seven bacterial suspensions were prepared from three independent cultures. 

#### 4.4.2. Determination of MIC

The procedure was modified from method described by Rahman *et al.* [35]. Briefly, pre-cultured *E. coli* and *E. coli* O157:H7 were diluted with M9 medium to 10^5^ colony forming unit/mL. The bacterial inoculum was then transferred (180 µL) into a 96-well microplate (NUNC ^TM^, Kamstrupvej, Roskilde, Denmark), followed by addition of 10 µL of freshly prepared CT solutions to yield final CT concentrations used in the assays, and 10 µL of 2,3,5-triphenyltetrazolium chloride (Sigma) as bacterial growth indicator (final concentration 25 µg/mL). Wells with live bacteria could be identified by the presence of a red deposit in the bottom of the incubation well. Preliminary tests showed no bacterial growth at concentrations of CT > 300 µg/mL. Therefore, MIC was determined over a range of concentrations from 0 to 300 µg/mL at 10 µg/mL intervals. Four replicate wells were prepared for each concentration of CT. Plates were incubated aerobically with horizontally shaking (120 rpm) at 37 °C for 12 h and visually examined for red deposit. The lowest concentration of CT that showed no red deposit at the bottom of incubation well was defined as the MIC. The experiment was repeated three times over two-week period.

### 4.5. Determination of the Effects of CT on OM Permeability and Cell Aggregation of E. coli

Since the MIC of both CT were similar across all strains of *E. coli* the model strain ATCC25922 [36,37,38] was used for subsequent experiments.

#### 4.5.1. Pre-culturing and Preparation of Bacterial Suspension

The bacteria were aerobically cultured (duplicate flasks) in M9 media containing 0 or 10 µg/mL of each CT at 37 °C for 10 h using the same procedure as in Section 4.4.1. The sub-lethal concentration of 10 µg/mL used in the pre-incubation was based on the MIC of PPC tannin (25–50% of MIC). In addition, bacteria was also aerobically cultured in two flasks with M9 media containing 0.05 µmol/mL of ethylenediaminetetraacetic acid (EDTA; Sigma) in the same manner as above for comparison since EDTA is a commonly used membrane permeabilizer. The bacterial cells suspensions that were used for the OM permeability were prepared in the same manner as described in Section 4.4.1, but using phosphate buffer. The bacterial suspensions were sampled and enumerated by dilution plating as described by Bach *et al*. [39].

#### 4.5.2. OM Permeability Assays

The OM permeability of *E. coil* prepared above was determined using a NPN uptake assay as described by Ikigai *et al.* [10]. The assay was conducted with black fluorotitre microplates (COSTAR^®^) with fluorescence being measured at 10 and 20 min after addition of NPN. Fluorescence of bacterial suspensions and phosphate buffer with NPN were also measured separately at the same time to estimate background fluorescence. The effect of CT on the OM permeability of *E. coli* was assessed by comparing the relative fluorescence value (RFV) of the two bacterial suspensions (with and without CT during pre-incubation). 

#### 4.5.3. Determination of Cell Aggregation by CT

##### 4.5.3.1. NPN Fluorescence Quenching Assay

Preliminary tests showed that the fluorescence intensity of mixture of bacterial cells suspension and NPN was decreased by addition of CT due to the aggregation of the cells. Therefore, the NPN assay described above was modified as described by Ikigai *et al.* [10] to estimate cell aggregation by measuring quenching of NPN fluorescence by CT. Briefly, 190 µL of bacterial suspension (pre-cultured without CT) as described above was transferred into wells of black fluorotitre microplates, and background fluorescence was measured. Ten µL of NPN (final concentration 10 µM) was then added to each well and fluorescence was measured. Freshly prepared CT solutions (10 µL) or EDTA (10 µL) were added to each well so as to result in a final concentration of 0, 50 or 200 µg/mL of CT or 0, 0.2, 0.4 or 1.0 µmol/mL of EDTA in respective wells. Fluorescence within each well was measured 10 min after CT and EDTA addition. Each assay was performed three times with three independent bacterial suspensions. Background fluorescence of the buffer + NPN and bacterial suspension were subtracted from the final fluorescence values.

##### 4.5.3.2. Fluorescence Imaging Analysis

The cell aggregation caused by CT was also examined using nucleic acid probes SYTO9 in the LIVE/DEAD® *Bac*Light Bacterial Viability kit (L7007, Molecular Probes) according to the manufacturer’s instructions [40]. SYTO9 stains all bacterial cells with green fluorescence. The assay procedure was similar to that for NPN fluorescence quenching assay described above, but the 10 µL of NPN was replaced with 1 µL of the SYTO9. The stained bacterial suspension (5 µL) was transferred onto glass slide 10 min after the addition of CT. The sample was covered with a coverslip and observed using Fluorescent Microscope2420A (2420A; Leica Microsystems, Wetzlar, Germany). 

### 4.6. Transmission Electron Microscopy Examination of Morphological Alteration of Cell Membrane

*E. coli* strain 25922 was incubated in M9 medium without CT (Control) or with 50 μg/mL of PPC or SF CT. Procedures for bacterial culture, inoculation and addition of CT into the culture were the same as described in Section 4.5.1. Phlorotannins isolated from brown seaweed (*Ascophyllum nodosum*) using a procedure described by Wang *et al.* [5] was included at the concentration of 25 µg/mL as positive control. The phlorotannins at this concentration have previously been shown to inhibit the growth of *E. coli* and *E. coli* O157:H7 and to affect their cell membrane structures [5]. Triplicate cultures were prepared for each tannins. After 12-h incubation, bacterial cells were harvested, processed and examined by TEM as described by Bae et al. [41] using an H-500 Hitachi TEM (Hitachi, Tokyo, Japan).

### 4.7. Data Calculations and Statistical Analysis

Amount of protein precipitated in experiment determining protein-precipitating capacities of PPC and SF CT was calculated as the difference between added protein and that present in the supernatant after CT addition. Data were fitted to a sigmoidal curve using nonlinear regression in Slidewrite Plus for Windows (version 7.01; Advanced Graphics Software, Inc., Rancho Santa Fe, CA, USA):

*y=a_0_*+*a/(1+exp(-(x-b)/c))*

where *y* = mg of protein (BSA or Rubisco) precipitated, *x* = µg of CT incubated, *a_0_+a* = estimated maximum amount (mg) of protein (BSA or Rubisco) precipitated, *b* = sigmoidal centre (mg of CT at the 50% of maximal protein precipitation), and *c* = sigmoidal width. The protein-precipitating capacity (*PP*) of each CT was expressed as the amount (µg) of the CT required to precipitate 1.0 mg of BSA or Rubisco protein.

The RFV obtained in experiments determining reactivity of PPC and SF CT with liposome, and effects of CT on OM permeability and cell aggregation of *E. coli* was calculated as the actual fluorescence value divided by 100 [27].

Data, except for that of MIC and TEM, were statistically analyzed by the MIXED procedure of SAS (SAS, Cary, NC, USA, 2009). Different CT at different concentrations in experiments other than MIC determination and TEM observation were arranged as completely random design. Replicates within each assay and replicate assays were considered random factors. Differences among treatments were tested using LSMEANS with the PDIFF option and adjusted by the Tukey’s test.

## 5. Conclusions

Condensed tannins from PPC possess greater antimicrobial activity against *E. coli* and *E. coli* O157:H7 than CT from SF. The greater anti-*E. coli* and anti-*E. coli* O157:H7 activity of PPC CT is attributed to their greater capacities to precipitate protein and cause cell aggregation. These properties lead to destabilization of OM structure and permeability. 
